# The Cambridge Structural Database

**DOI:** 10.1107/S2052520616003954

**Published:** 2016-04-01

**Authors:** Colin R. Groom, Ian J. Bruno, Matthew P. Lightfoot, Suzanna C. Ward

**Affiliations:** a Cambridge Crystallographic Data Centre, 12 Union Road, Cambridge CB2 1EZ, England

**Keywords:** Cambridge Structural Database, CIF archive, open data, crystal structure database

## Abstract

The Cambridge Structural Database (CSD) contains a complete record of all published organic and metal–organic small-molecule crystal structures. The database has been in operation for over 50 years and continues to be the primary means of sharing structural chemistry data and knowledge across disciplines. As well as structures that are made public to support scientific articles, it includes many structures published directly as CSD Communications. All structures are processed both computationally and by expert structural chemistry editors prior to entering the database. A key component of this processing is the reliable association of the chemical identity of the structure studied with the experimental data. This important step helps ensure that data is widely discoverable and readily reusable. Content is further enriched through selective inclusion of additional experimental data. Entries are available to anyone through free CSD community web services. Linking services developed and maintained by the CCDC, combined with the use of standard identifiers, facilitate discovery from other resources. Data can also be accessed through CCDC and third party software applications and through an application programming interface.

## Abstract 2

This paper is the definitive article describing the creation, maintenance, information content and availability of the Cambridge Structural Database (CSD), the world’s repository of small molecule crystal structures.

## 1. The value of sharing crystal structures   

The ongoing stewardship of the Cambridge Structural Database (CSD) has been the core activity of the Cambridge Crystallographic Data Centre (CCDC) since its inception in 1965. The CCDC is committed to providing a permanent archive of crystal structures and making these available to all. This non-profit, charitable organization is overseen by an international board of trustees drawn from the community it serves.

The CSD contains all published organic and metal–organic small-molecule crystal structures whose structures have been determined using the technique of crystallography. In addition, it acts as a publication vehicle for structure determinations with no accompanying manuscript.

Specifically, the CSD contains both X-ray and neutron diffraction analyses from a single-crystal study or a powder study where cell parameters, atomic coordinates and refinement are reported. To ensure comprehensive coverage of single-crystal data, cell parameters and all available data are included even if no coordinates are available. Powder structures without coordinates are available from the International Centre for Diffraction Data (ICDD; Kabekkodu *et al.*, 2002 ▸).

The CSD covers all organic and metal–organic structures, where organic is generally taken to mean a carbon-containing molecule. The CSD also contains boron compounds containing one or more B—H or B—OH bond and borazines and ring compounds containing any two of the following elements: N, P, S, Se and Te. Purely inorganic structures that do not fit the criteria above are added to the Inorganic Crystal Structure Database (ICSD; Belsky *et al.*, 2002 ▸) produced by FIZ Karlsruhe or the Metals Database (CRYSTMET; White *et al.*, 2002 ▸) for metals and alloys. Peptides and polysaccharides of up to 24 residues and mono-, di- and tri-nucleotides are included in the CSD, higher oligomers are covered by the Nucleic Acids Database (Coimbatore Narayanan *et al.*, 2014 ▸) with the Protein Data Bank (PDB; Berman *et al.*, 2000 ▸) curating and sharing structural data of larger biological macromolecules. In all cases, these guidelines are relaxed where there is clear scientific merit in including a structure in multiple resources.

The database provides value in two distinct ways. The first simply relates to the aggregation and standardization of structures, which facilitates access to individual entries. This brings value both to the data generators and consumers. A single archive of all structures allows crystallographers to avoid the inadvertent redetermination of structures and provides a mechanism by which they can archive the output of their work at a specialist data centre for their own future use. Of course, it also allows for the easy sharing of their work, massively increasing their sphere of influence. Such sharing has always been the norm for the crystallographic community and the use of this worldwide, standard, specialist discipline repository allows individuals to demonstrate their adherence to the new data sharing mandates of various funding bodies. As all entries are subject to both automatic and manual curation, they can usually be used without further processing. Indeed, one might argue that the financial cost of maintaining such a resource, although significant, is recovered many times over by removing the need for repeated correction by users.

A further vital property of the CSD is its comprehensive and up-to-date nature. As it represents the complete record of published structures and is updated within a few moments of a new publication, users can have confidence that there are no published structures of relevance of which they are unaware.

The second distinct benefit of the database comes from the study of the *collection* of entries. This was perhaps best articulated by the founder of the CSD, Dr Olga Kennard, who, recounting a discussion with JD Bernal commented, ‘We had a passionate belief that the collective use of data would lead to the discovery of new knowledge which transcends the results of individual experiments’ (Kennard, 1997 ▸). The motivations behind the determination of crystal structures do differ, the most common probably being the confirmation of a molecule’s chemical identity. However, the use they are put to once in the database usually bears no similarity to these motivations. Two illustrative examples are perhaps the establishment of the ability of C—H groups to act as hydrogen-bond donors (Taylor & Kennard, 1984 ▸) and ‘structure correlation’ – the linking of three-dimensional geometry to reaction pathways (Bürgi & Dunitz, 1983 ▸, 1997 ▸).

This ‘new knowledge’ relates primarily to the geometry of molecules and the interactions they make. A knowledge of these factors underpins huge areas of both fundamental and applied science (Wong *et al.*, 2010 ▸). They form the basis of our understanding of the energetics of molecular conformation – from bond lengths and angles, through to torsional preferences. They also teach us about the fundamentals of molecular recognition, be it small molecules interacting with small molecules in a lattice or with a protein. Such appreciation is vital for materials sciences and pharmaceutical research and development. Perhaps the most quantitative assessment that can be made of the value of the resource is that the previous published description of the CSD (Allen, 2002 ▸), which this article supersedes, has received over 10 000 citations.

## 2. The Cambridge Structural Database   

**Figure 1:**
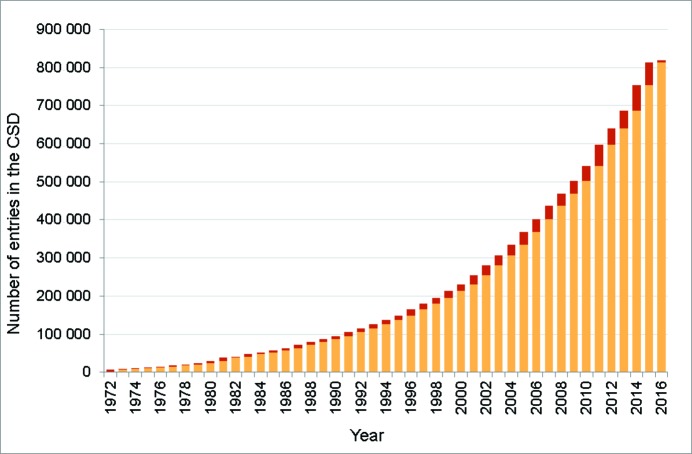
Growth of the CSD since 1972, the red bar shows structures added annually.

**Figure 2:**
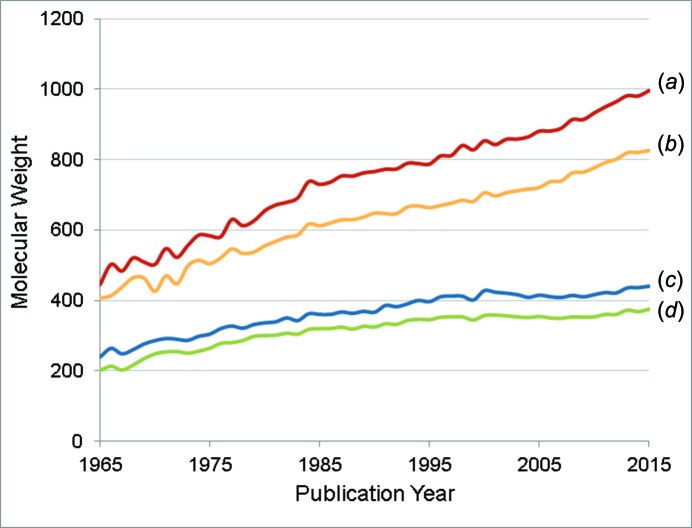
The increase in the average molecular weight of CSD entries since 1965, with (*a*) average formula weight per *Z*′ of metal–organic structures, (*b*) average molecular weight of heaviest component of metal–organic structures, (*c*) average formula weight per *Z*′ of organic structures and (*d*) average molecular weight of the heaviest component of organic structures.

**Figure 3:**
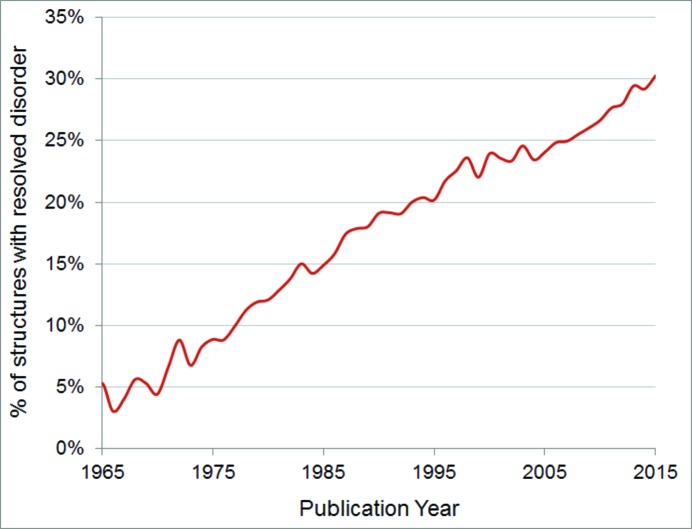
The increase in resolved disorder in CSD entries since 1965.

In 2015 the number of entries in the CSD surpassed 800 000 (Fig. 1 ▸). This is twice the number of entries in the database less than a decade ago. Comparing statistics based on the database as it was then allows us to see what has changed in the last decade – and what has not. Table 1 ▸ shows that the proportion of structures which are organic or metal–organic structures (which we classify as structures containing a transition metal, lanthanide, actinide, or Al, Ga, In, Tl, Ge, Sn, Pb, Sb, Bi, Po) has remained fairly constant. What has changed is the complexity of the structures being published: the average number of atoms per structure and the average molecular weight have increased (Fig. 2 ▸), as has the proportion of structures that are polymeric or that have resolved disorder (Fig. 3 ▸).

Another significant change is in the number of new structures published per year, which in 2015 was almost twice the number published during 2006. Structures in the CSD are associated with over 400 000 articles from 1600 publication sources. Table 2 ▸ shows the top 20 publication sources currently represented in the CSD; these account for 67% of the entries in the database.

The increase in volume and complexity of structures deposited into the CSD over the past decade has presented both administrative and scientific challenges. This article will describe how these have been addressed, but to do this we must first look back at the journey that led us to where we are today.

## 3. The development of the CSD   

What today we call the Cambridge Structural Database began life as ‘a computer-based file containing both bibliographic information and numerical data abstracted from the literature and relevant to molecular crystal structures, as obtained by diffraction methods’ (Kennard & Watson, 1970*b* ▸). Work compiling this file began in 1965 and contents were made available through the series of printed volumes, ‘*Molecular Structures and Dimensions*’ (Kennard & Watson, 1970*a* ▸). Over time the file developed into a more structured database and the software used to generate and check data evolved into interactive applications enabling chemical searching and analysis of three-dimensional structural data. Together these provided the foundations for the rich suite of CSD-based applications available today.

Just as the database and software evolved so too did the way in which crystal structure data is communicated. In the early days atomic coordinates were published as tables in scientific articles. These were transcribed manually for inclusion in data files and databases. In the 1990s, a file format called CIF (now the Crystallographic Information Framework) was proposed as a standard for the interchange of crystallographic data. CIF is now ubiquitously used to capture the results of a diffraction experiment and enables streamlined publication of results alongside journal articles and in data repositories (Hall *et al.*, 1991 ▸; Brown & McMahon, 2002 ▸; Bernstein *et al.*, 2016 ▸; Hall & McMahon, 2016 ▸).

Advances in science and technology have enabled many of the processes involved in generating and maintaining a database to be automated. However, in order to ensure accuracy and quality, the oversight and input of expert scientists remains as much a requirement today as it did in the early days. Of particular importance is the assignment of rich metadata, including the chemical identity of the substance studied during the diffraction experiment. Without this, the ease with which the data can be reused is greatly limited. Subsequent sections of this paper describe our informatics systems that combine technology and expertise in a way that minimizes the cost of curation without compromising on the reusability of the data.

The increasing digital availability of the basic results of a diffraction experiment has also had an impact on the overall nature of the CSD. Originally, it might best have been described as a secondary resource of data abstracted from the literature. Today, new source data files are almost always deposited directly into the CSD making it much more of a primary data resource. Indeed, many structures are published only and directly through the CSD as CSD Communications (previously known as Private Communications). Whilst the CCDC has never charged for deposition, curation, archiving or for access to the primary source data files, early versions of the CCDC’s informatics systems did have technical barriers that hindered access to structures by researchers. Here, we describe developments that ensure entries from the CSD are made available to the widest possible community through CCDC services and third party resources.

## 4. Deposition and retrieval of data   

The method for deposition into the CSD has evolved since the advent of the CIF format in the 1990s, when email depositions dominated. In 2009 the CCDC launched an online web-based tool which is now the main route for deposition. In 2015, 90% of structures were deposited with the CCDC prior to publication and 85% were submitted through this service. A key benefit of this early deposition is that at this point the crystallographer who generated the data is likely to be the depositor and be in a position both to provide the richest data and to respond to any issues most effectively.

During deposition, the CIF syntax is automatically checked based on the checks in *enCIFer* (Allen *et al.*, 2004 ▸) and the depositor is required to fix any issues before continuing with the deposition process. Depositors are strongly encouraged to deposit structure factors in line with the IUCr’s publication standards for crystal structures (IUCr, 2011 ▸). The embedding of reflection data into the CIF by structure refinement programs such as *SHELXL* (Sheldrick, 2015 ▸) greatly simplifies this process. As a consequence the amount of reflection data stored at CCDC has increased significantly and the majority of new depositions now include this data.

**Figure 4:**
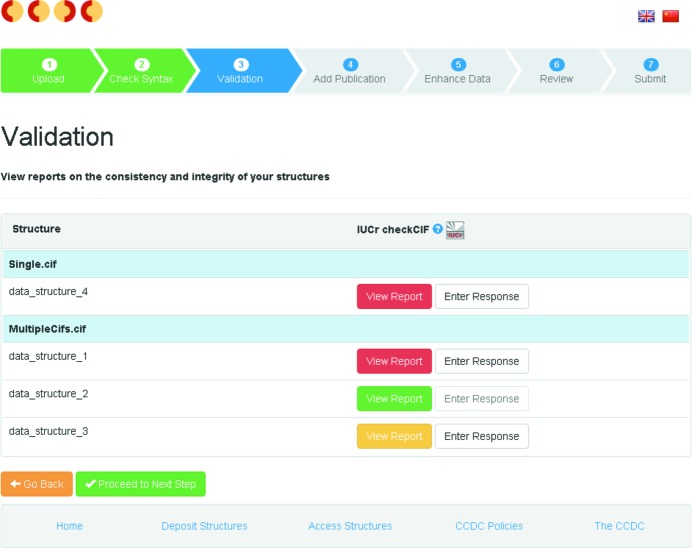
The validation step of the CSD Deposition process showing the integration with *checkCIF* (CCDC, 2016*b* ▸).

In 2015 the *checkCIF*/*PLATON* service (Spek, 2009 ▸) was integrated into the CCDC’s deposition process (Fig. 4 ▸). This allows the researcher to generate validation reports and embed validation responses into the ‘structure of record’ during deposition to the CSD. Reviewers and publishers can read the *checkCIF* report alongside the deposited data to aid peer review of submitted papers.

During deposition, key metadata are extracted from the CIF and presented to the researcher giving them the opportunity to check and further enhance the data that is shared through the CSD. To aid with this process a three-dimensional representation of the structure is displayed to the depositor using *JSmol* (Hanson *et al.*, 2013 ▸).

On deposition, each dataset is assigned an accession identifier referred to as a ‘CCDC number’ in the format CCDC 1234567 (older entries have six digits). This uniquely identifies the data associated with a particular structure determination and persists for the lifetime of the dataset. CCDC numbers are communicated to the depositor once it has been confirmed that the dataset is not a duplicate submission, usually within seconds of deposition. Should the data require further investigation, CCDC Deposition Coordinators address any problems, minimizing the delay in providing the CCDC number to the depositor. A reference code (known as a CSD refcode and in the format ABCDEF) is assigned to structures as they are indexed into the CSD itself. Where possible, determinations of the same substance are assigned into a CSD refcode family (with a CSD refcode format of ABCDEF01). These codes are used as a common way of referring to structures extracted from the CSD-System.

CCDC numbers are used in manuscripts to indicate the location of the data that supports results described in that article. They are used as the basis for providing links directly to the structure from within the article when published. Prior to publication, data is stored in a confidential data archive and is only available to referees and publishers during the peer review process. Providing access to the structure of record, reflection data and validation reports helps ensure the accuracy and integrity of published science. Authors and depositors are able to revise data stored at CCDC up to the point of publication and retain a consistent CCDC number. About one quarter of all submissions are revised during the deposition process. In 2015 alone, over 67 465 unique CCDC numbers were assigned and data was deposited by over 10 000 unique depositors.

For structures associated with a journal article, computational workflows and processes with the major publishers handle the flow of data during the publication process. The publication of an article referencing a dataset results in the immediate public release of the corresponding structure. This is automatically triggered by feeds from the publisher or, failing that, by the identification of a manuscript with a CCDC number. Structures deposited with the intention of accompanying a publication are held securely in trust for a period of 1 year. If no publication is identified within that year, authors are contacted to confirm that the structure should be published as a CSD Communication. If the structure is still intended for another publication then the embargo period is extended for another year.

A structure published as a CSD Communication is freely available through the CSD within seconds of CCDC number assignment. As the general appreciation of the value of data sharing has increased so has the popularity of publishing structures directly through the CSD. In fact, although only 2% of structures in the CSD are CSD Communications, 2015 saw over 4400 CSD Communications published, making it likely that this will soon become the most popular way in which to publish crystal structures.

**Figure 5:**
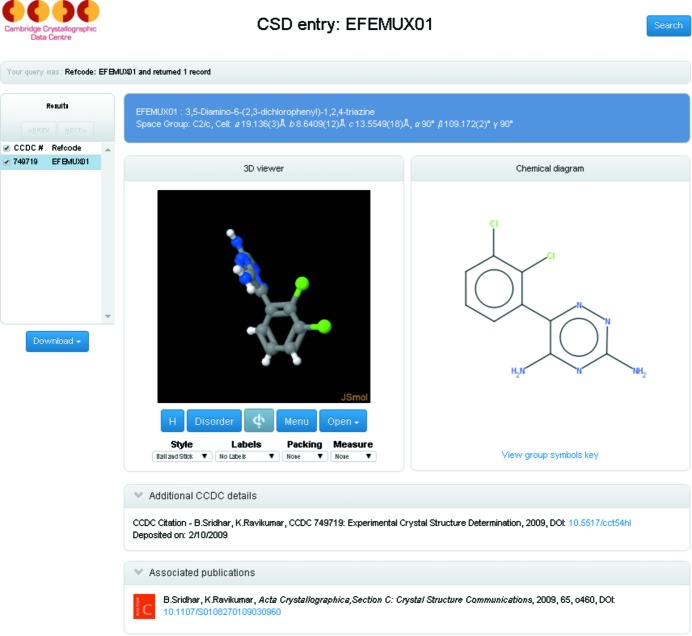
Screenshot of the CSD community Access Structures results page. This is the ‘landing page’ for many referring services based, for example on DOI or CCDC number (CCDC, 2016*a* ▸).

At the point of publication, entries are available to anyone through free CSD-Community web services. These services allow anyone to access published structures either *via* the CCDC website or by following links from other resources. They provide an interactive visualization of the three-dimensional structure through *JSmol* (Hanson *et al.*, 2013 ▸), a two-dimensional chemical diagram and key metadata associated with the entry (Fig. 5 ▸). Individual data files can also be downloaded and used by anyone wishing to investigate or explore the entries in more detail.

## 5. The creation of the CSD   

### 5.1. Processing entries   

**Figure 6:**
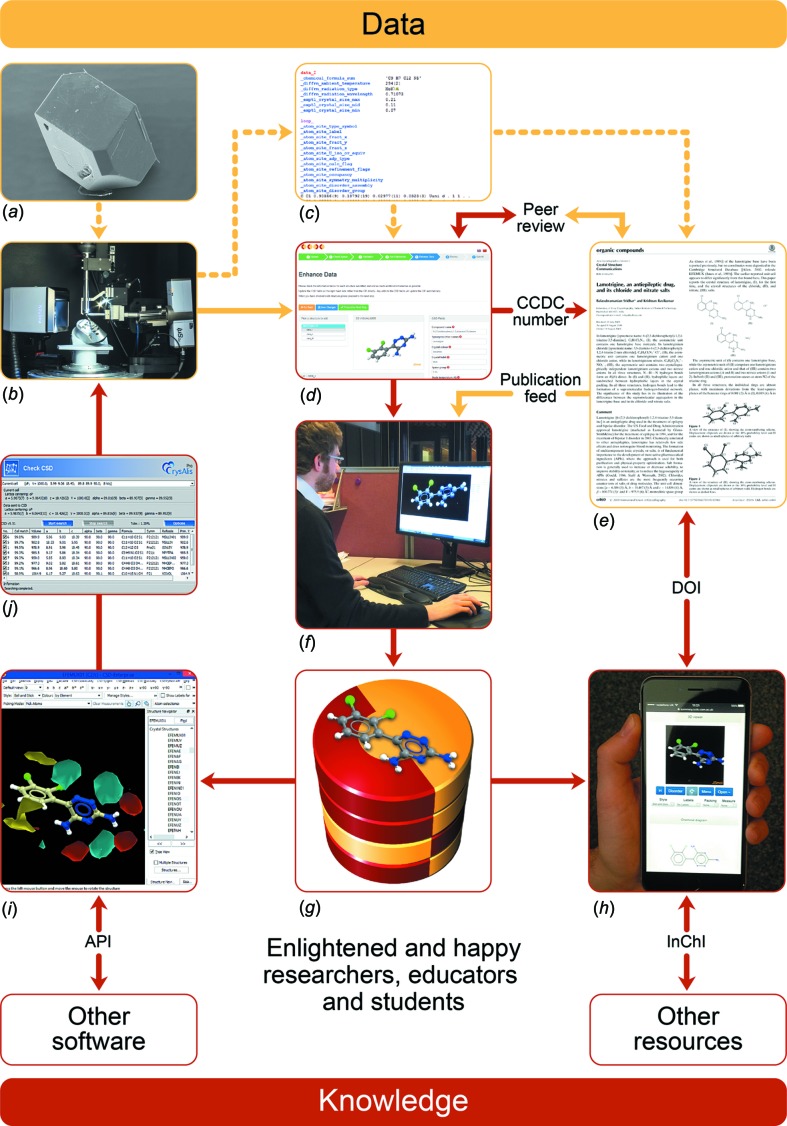
An illustration of the flow from sample to CSD entry. Solid lines are the elements of the process involving the CCDC. A crystalline sample (*a*) is put on a diffractometer (*b*) for an experiment that ultimately results in the experimentally determined coordinates of atoms in the crystal that are captured in a CIF file (*c*). The CIF file is deposited with the CCDC (*d*) and may be associated with a scientific article (*e*). On publication of the article, the structure is validated and enriched by CCDC editorial software and staff (*f*) to create an entry in the CSD (*g*). Structures are publicly accessible through CSD community services such as Access Structures (*h*). Standard identifiers such as DOIs and InChIs facilitate links between articles, structures and other resources. Software developed by the CCDC (*i*) enables the knowledge embedded in the CSD to be applied to a range of scientific problems including aiding in the determination and refinement of future structures through free resources such as CellCheckCSD (*j*). The flow of data is largely represented using lamotrigine (Sridhar & Ravikumar, 2009 ▸), the 500 000th entry in the CSD, CSD refcode: EFEMUX01; CCDC number: CCDC 749719; DOI: 10.5517/cct54hl. Image (*a*) is a crystal of *p*-aminobenzoic acid (Sullivan & Davey, 2015 ▸) courtesy of Rachel Sullivan and Roger Davey, University of Manchester, CSD refcode: AMBNAC10; CCDC 983122; DOI: 10.5517/cc1200mq. Image (*b*) courtesy of Andrew Bond, University of Cambridge.

Due to the rising number of structures, depositions and transactions a new processing system, *CSD-Xpedite*, has been developed, automating most informatics processes required to manage depositions and process crystal structures into entries in the CSD. Fig. 6 ▸ shows how data flows through the system and where various users interact with the system.

*CSD-Xpedite* is built around Microsoft *Dynamics CRM* (Microsoft, 2016*a* ▸) and *SharePoint* (Microsoft, 2016*b* ▸). The system is designed to store all data in one unified system and many automated processes have been incorporated to reduce the number of manual interventions required to process entries through the CSD creation process. This scalable system allows for the ever increasing rate of deposition, and its modular nature allows extra functionality to be incorporated with minimal disruption. One important aspect of *CSD-Xpedite* is the use of a new extensible file format for internal storage of CSD entries. This format allows a fuller representation of the underlying data than in previous formats (BCCAB, ASER). Databases for use in the CSD-System are created from this master database.

A key component in *CSD-Xpedite* is *CSD-Editor*, an interactive tool for processing structures such that they can enter into the CSD. This uses many of the visualization and menu options available in the CSD-System program *Mercury* (Macrae *et al.*, 2006 ▸). Deposited CIFs can be easily viewed alongside any associated article. Errors and warnings are displayed for each structure to allow expert structural chemistry editors to concentrate their efforts on the challenging scientific parts of the process.

### 5.2. Assignment of chemical identity   

An important aspect of creating the CSD is the assignment of the chemical representation to structures so that these can be reliably searched and analysed using structure-based methodologies such as substructure search. In the past, the chemical representation was assigned by scientific editors visualizing the structure and consulting any associated article. A program called *deCIFer* (Bruno *et al.*, 2011 ▸) now helps automatically assign ‘chemistry’ to structures.

*DeCIFer* uses the information already in the CSD to interpret a new structure and add a chemical representation to the atomic coordinates in the CIF. The stages involved in the automatic creation of the CSD entry include resolution of any disorder in the structure, detection of bonds and determination of bond types and charges. Assignment of bond types, charges and the inference of missing H atoms uses a probabilistic Bayesian approach which allows all the existing entries in the CSD to be used to help assign the chemistry to new entries.

The final step of the deCIFer process is to validate the assignment by looking at improbable features such as unprecedented bond types, unlikely oxidation states, unlikely metal–metal bonds and other empirical indicators such as non-planar double bonds. From this analysis a reliability score is calculated which highlights any possible errors in the representation of the structure which can then be reviewed and corrected during CSD entry validation. It is important to note that this reliability score simply gives a confidence value to the automatic treatment of the entry; this is not necessarily related to the ‘quality’ of the structure.

With ‘chemistry’ assigned, an internally developed diagram generation algorithm takes the three-dimensional coordinates of an entry and ‘flattens’ these, producing a two-dimensional representation with minimal overlaps of atoms and bonds. This procedure also takes advantage of the many two-dimensional diagrams already available for related entries, often drawn previously by scientific editors, which increases both the quality and consistency of diagrams.

Although the automatic deCIFer process is used as much as possible structures are still manually viewed by expert structural chemists before they are added to the CSD.

## 6. Using the CSD   

### 6.1. Identifying and linking digital objects   

At the point a structure can be made public a Digital Object Identifier (DOI) is associated with the deposited dataset (580 000 are currently available). This DOI allows a third party to link to a structure summary page without needing to know details of linking services provided by the CCDC. In addition the DOI provides the basis for a more formal citation of the deposited dataset in line with the spirit of the Joint Declaration of Data Citation Principles (Data Citation Synthesis Group, 2014 ▸). The metadata provided to the DOI registration agency DataCite (DataCite, 2016 ▸) indicates key elements of a dataset citation including contributor, a title and publication year. This enables citation of the dataset independently of the associated article and thus helps ensure recognition of the specific contribution made by the crystallographer, who may not always be included in the article’s author list.

Metadata submitted by the CCDC to DataCite when generating a DOI is openly accessible and facilitates interoperability with third party systems to improve the discoverability of data. Examples where other parties have taken advantage of this include the Thomson Reuters Data Citation Index (Thomson Reuters, 2016 ▸), where data is currently available for 530 000 CSD entries, and the prototype RDA/WDS Data-Literature Interlinking service (Burton *et al.*, 2015 ▸). In order to support this interoperability, additional metadata items are made public including the DOIs of associated articles and the chemical name of the substance studied.

Discoverability of data by chemists and biologists is enabled by establishing links to datasets from services such as ChemSpider (Pence & Williams, 2010 ▸) and PubChem (Bolton *et al.*, 2008 ▸). The overlap between these resources and the CSD is identified by taking advantage of the International Chemical Identifier Standard (InChI) which provides a unique and canonical representation of the chemical substance studied (Heller *et al.*, 2015 ▸). Links from ChemSpider and PubChem have been established for over 52 000 compounds that could be reliably identified using InChIs as being in common between these resources and the CSD. InChIs have also been used to identify correspondences between CSD entries and ligands bound to macromolecules in structures archived in the Protein Data Bank (PDB; Berman *et al.*, 2000 ▸). This lookup is enabled by a CCDC web service that identifies the best-representative CSD entry for a given molecule and provides access to its coordinates. This is a particularly useful resource for structural biologists refining or investigating the structure of a protein ligand. Representative structures are freely available for approximately 1500 PDB ligands in a Chemical Component Model file provided by the PDB (wwPDB, 2015 ▸).

### 6.2. Accessibility and efficiency   

The CSD community web services detailed earlier provide free access to the entire collection of structures. As well as these services there are a number of other avenues to explore and exploit the data ranging from free lookup tools such as *CellCheckCSD* (Wood, 2011 ▸) to advanced search, analysis and validation tools in the CSD-System (Bruno *et al.*, 1997 ▸, 2002 ▸, 2004 ▸; Macrae *et al.*, 2008 ▸). More specialist applications are provided as part of the CSD-Enterprise suite.

*CellCheckCSD* is an automated tool which uses the data from the CSD to check unit cells during data collection and can be used to check that the structure is novel rather than the starting material, a by-product or another previously determined structure.

The CSD-System enables knowledge to be gained from the collection of data through powerful two-dimensional/three-dimensional search capabilities, extensive geometry analysis tools, inter- and intramolecular interaction analysis, generation of high impact graphics and the ability to delve further into the data using a Python-based applications programming interface.

Visualizing three-dimensional structural data can be a powerful way to teach chemistry concepts (Battle & Allen, 2012 ▸; Henderson *et al.*, 2011 ▸; Battle *et al.*, 2011 ▸, and references therein). Through the CSD community services, educators worldwide are able to access all 800 000 entries in the CSD, but the enormity of the database means it is not a simple process to identify the key structures that are most appropriate for the class. To this end a CSD teaching database (Battle *et al.*, 2010 ▸) has been compiled. This is a collection of around 700 carefully selected crystal structures, targeted to represent a diverse range of chemistry and to allow teachers to demonstrate key chemical concepts and principles.

To support the free archiving services and free universal access to all individual structures, users of client installed software (*i.e.* the CSD-System) are asked to contribute to the running costs of the CCDC. In many cases this is done at a national level, allowing unfettered use, with the modest contribution levels set according to their economic status. These contributions fund the maintenance of the CSD, whereas larger contributions from for-profit users of the system fund its ongoing development (Bruno & Groom, 2014 ▸).

## 7. Future   

Since its very inception, X-ray and neutron crystallography has been the method of choice for the elucidation of the full three-dimensional structure of molecules (Wilkins, 2013 ▸). However, techniques involving electron diffraction (see for example Yun *et al.*, 2015 ▸), atomic force microscopy (see for example Gross *et al.*, 2009 ▸), free electron lasers (Barty *et al.*, 2013 ▸) and NMR crystallography (Baias *et al.*, 2013 ▸) have already shown their potential. In some cases, this will involve the capture of molecular structures not in a crystalline lattice, so systems have been designed to allow for this. Developments are also already underway to allow effective treatment of predicted crystal structures (Reilly *et al.*, 2016 ▸).

Although the CSD contains all *published* crystal structures, it has been estimated that only 15% of structures *determined* are published (UK National Crystallography Service, 2014 ▸). Automatic links in software used during structure determination (Dolomanov *et al.*, 2009 ▸), the ease with which structures can be deposited, attribution of credit in the form of a DOI and continued demonstration of the value to science of depositing crystal structures (Berman *et al.*, 2015 ▸) may help close this gap.

We expect to see a continued push towards the federation of databases containing molecular structures (Sali *et al.*, 2015 ▸) and the development of links between repositories. As these individual repositories grow, methods for searching them will also need to be improved.

There is now an expectation in science that data is freely, openly and instantly available. With modern informatics techniques this is trivial to achieve, but is often done at the expense of data quality, completeness, integrity and with questionable sustainability. History has shown us that all these are best achieved by specialist data centres, maintaining specialist data repositories. The challenge of finding a universally agreed ‘perfect’ funding model, if one exists, remains (Bruno & Groom, 2014 ▸).

The authors of this article were unable to write a concluding paragraph that expresses the continued value of the CSD better than those who had the inspiration to found it, Olga Kennard and J. D. Bernal (1901–1971). We therefore conclude with an excerpt from a historical memoir presented in 1995. ‘*I think that the great ocean of truth is still in front of us and that we will continue to discover new aspects of this truth. Some of them will be discovered through the insight of outstanding individuals and some through the insight which Bernal predicted could be gained by more ordinary mortals through the analysis and transformation of the pebbles of information which have accumulated over the past decades. We have the tools and resources to do this and Bernal’s inspiration is still with us*’ (Kennard, 1995 ▸).

## Figures and Tables

**Table 1:** Development of the CSD over a 10-year timespan from 2006 to 2015

	2006	2015
Number of CSD entries	400 374	800 239
Number of compounds	363 372	731 675
Number of associated articles	232 858	408 899
New entries	34 030	60 122
Entries classed ‘Organic’	43%	43%
Entries with *R*-factor < 10%	92%	94%
Average atoms per structure	68.6	80.6
Polymeric entries	7%	11%

**Table 2:** Publication sources for CSD entries

Journal (Publisher)	% CSD
*Inorg. Chem.* (ACS)	8
*Dalton Trans.* (RSC)	6
*Organometallics* (ACS)	6
*J. Am. Chem. Soc.* (ACS)	5
*Acta Cryst. Section E* (IUCr)	5
*J. Organomet. Chem.* (Elsevier)	3
*Chem. Commun.* (RSC)	3
*Acta Cryst. Section C* (IUCr)	3
*Inorg. Chim. Acta* (Elsevier)	3
*Chem. Eur. J.* (Wiley)	3
*Polyhedron* (Elsevier)	3
*Angew. Chem. Int. Ed.* (Wiley)	3
*Eur. J. Inorg. Chem.* (Wiley)	2
*J. Org. Chem.* (ACS)	2
*CrystEngComm* (RSC)	2
*Cryst. Growth Des.* (ACS)	2
*Acta Cryst. Section B* (IUCr)	2
*CSD Communications* (CCDC)	2
*Z. Anorg. Allg. Chem.* (Wiley)	2
*Tetrahedron* (Elsevier)	2
